# On the Alleged Increase of Cancer

**Published:** 1893-05-20

**Authors:** George King, Arthur Newsholme


### ON THE ALLEGED INCREASE OF CANCER.

By George King, F.I.A., F.F.A., and Arthur Newsholme, M.D., M.R.C.P. Communicated by Dr. J. S. Bristowe, F.R.S. Received February 27th, 1893.

(We are indebted to the author for the following important communication to the Royal Society.)

*Abstract.*

Attention is first drawn to the alarming increase in mortality from cancer, shown by the Registrar-General's figures, and to the fact that the view that this increase is due to more accurate diagnosis and certification has been partially abandoned.

An attempt is then made to test this conclusion, by a study from an independent standpoint of the official cancer death-rates for England and Wales, Scotland, and Ireland; and by a comparison of these death-rates with other data obtained from the experience of the Scottish Widows' Fund Life Assurance Society, and from the official cancer returns for the city of Frankfort-on-the-Main.

In order to make the figures from these different sources exactly incomparable, corrections have been made for variations in age and sex distribution. A standard population is taken (that of the "English Life Table, No. 3. Persons".) The death-rates at the different age-groups in each case are then multiplied into the populations at the corresponding age-groups in the standard population assumed as a common basis. Thus we obtain in each case the total deaths from cancer per annum a million persons aged 25 and upwards, grouped as in the standard population, and can contrast the different totals obtained, without any fallacy arising from varying age and sex distribution of population.

The results obtained are grouped in septennial periods, as the figures relating to the Scottish Widows' Fund Assurance Society were only obtainable in septennial periods. From these septennial results, the corresponding death-rates are obtained for each single year by an application of the graphic method employed by Milne in the construction of his Carlisle Life Table. These are shown as a series of curves.

The Irish curves are the lowest, probably because medical attendance in Ireland owing to poverty is on the average more meagre than in Great Britain. The English curves for males and females are very far apart. The Scottish curves for the two sexes are nearer together than the English, the apparent cancer mortality in Scotland for males being higher and for females lower than in England. The greater propinquity of the Scotch male and female curves may be ascribed to more correct diagnosis and certification in Scotland than in England. This view does not, however, explain why the female English is higher than the female Scotch curve; and it must be assumed, therefore, that there is some condition more favourable to the causation of cancer in English than in Scotch female life.

The Scottish Widows' Fund curve has the easiest gradient of all, probably pointing to more accurate diagnosis and certification than for the whole country, especially at the earlier periods.

That the apparent increase of cancer is at any rate chiefly due to improved diagnosis is shown by a comparison of the male and female curves respectively. They run practically parallel throughout. If cancer had really increased, its increase would probably have been an approximately equal percentage in the two sexes, and consequently the curves would have widened their distance apart. Even if—assuming that a real increase of cancer had occurred—the increase were unequal in amount in the two sexes, it is highly improbable that the increase would have been of such a distribution as to maintain the parallelism of the male and female curves.

The statistics for Frankfort-on-the-Main enable us to classify cancer in accordance with the part of the body primarily affected. We have, therefore, classified the returns into two groups, according as the cancer is "accessible" or easy of diagnosis, and "inaccessible" or difficult of diagnosis. The results of this classification show that in those parts of the body in which cancer is easily accessible and detected there has been no increase in cancer mortality between 1860 and 1889. It is true that the majority of deaths from "accessible" cancer are among women—the deaths from "accessible" cancer among men at Frankfort-on-the-Main being too few to be, when considered alone, trustworthy—but we know of no reason for supposing that, while female cancer of "accessible" parts has remained stationary, male and female cancer of the other parts of the body has really increased.

The general conclusions arrived at are that—

1. Males and females suffer equally from cancer in those parts of the body common to man and woman, the greater prevalence of cancer among females being due entirely to cancer of the sexual organs.

2. The apparent increase in cancer is confined to what we have called inaccessible cancer. This is shown (*a*) by the Frankfort figures; (*b*) by the fact that the difference between the rates for males and females respectively is approximately constant, and does not progressively increase with the apparent increase in cancer in each of the sexes; and (*c*) because the apparent increase in cancer among the well-to-do assured lives, who are presumably attended by medical men of more than average skill, is not so great as among the general population.

3. The supposed increase in cancer is only apparent, and is due to improvement in diagnosis and more careful certification of the causes of death.

